# Choroidal thickness in relation to diopter and axial length among myopic children

**DOI:** 10.3389/fmed.2023.1241352

**Published:** 2023-10-20

**Authors:** Huailin Zhu, Changyang Liu, Mingjun Gao, Siqi Zhang, Lan Zhang, Qi Zhao

**Affiliations:** Department of Ophthalmology, The Second Hospital of Dalian Medical University, Dalian, China

**Keywords:** choroidal thickness, optical coherence tomography, myopia, spherical equivalent refraction, axial length

## Abstract

**Purpose:**

The aim of this study was to analyze the relationship between diopter (D) and choroidal thickness in myopic children by describing the values of choroidal thickness, and explore choroidal thickness as an important indicator for evaluating the progression of myopia.

**Methods:**

The study included myopic and emmetropic children aged 6–14 years (156 eyes) from the Second Hospital of Dalian Medical University. The participants were divided into four groups according to the spherical equivalent refraction (SER): low myopes, moderate myopes, high myopes, and emmetropes. Choroidal thickness was measured in nine areas using the Early Treatment Diabetic Retinopathy Study (ETDRS) grid layout, which divided the areas into concentric circles of 0 × 0 mm, 3 × 3 mm, and 5 × 5 mm from the Sub Fovea. The images were obtained manually with spectral-domain optical coherence tomography scanner.

**Results:**

There were significant differences of choroidal thickness in the nine areas of the ETDRS grid for all myopes. The distribution of choroidal thickness in low and moderate myopes were different from that in high myopes. In the horizontal direction, choroidal thickness decreased from the temporal to nasal areas for all myopes. In the vertical direction, the choroidal thickness in the perifovea was smaller than that in the parafovea (SER ≥ −2.75D), and the choroidal thickness in the perifovea was greater than that in the parafovea (SER < −2.75D). When comparing emmetropes with myopes, the closer the choroidal thickness was to the central fovea, the more significant the differences were, while the differences were smaller to the perifovea. Among all children, choroidal thickness was positive to SER and negative to axial length (AL) in all areas. For every 1D increase in myopia, the subfoveal choroidal thickness decreased by 13 μm, and for every 1 mm increase in AL, the subfoveal choroidal thickness decreased by 23 μm. Furthermore, SER and AL showed the strongest correlation with choroidal thickness in the inferior area.

**Conclusion:**

Optical coherence tomography results revealed choroidal thickness was thinner in myopic children. Choroidal thickness was positive to SER and negative to AL. Therefore, we consider choroidal thickness to be an important indicator for evaluating the myopia progress.

## 1. Introduction

Myopia is a common ocular condition characterized by excessive refractive power of the lens and cornea or elongation of the eyeball (1), so that when the eye is relaxed, parallel light rays pass through the eye’s refractive system and focus on the front of the retina, thus distorting the image on the retina (2). Statistics reveal that over 400 million people worldwide are affected by myopia, with more than 90% concentrated in Asian countries. Myopia has shown a trend of increasing prevalence, particularly among the younger population (3–5). The World Health Organization predicts that, half of the global population may be myopic, with children being the most affected by 2050 (6). A study conducted in 2020 found that during the COVID-19 pandemic, the combination of home confinement, reduced outdoor activities, and increased screen time significantly contributed to a greater increase in myopia progression among children (7–10). Various indicators, including choroidal thickness, refractive error, and axial length (AL), can be used to evaluate the development of myopia. Among these indicators, choroidal thickness plays a more crucial role in regulating ocular growth and myopia development in children and adolescents compared to spherical equivalent refraction (SER) and AL, which are indirect assessment measures (11, 12).

The choroid is a thin, smooth, elastic, and highly vascular brown membrane located between the retina and sclera within the eye. Its primary functions include nourishing the outer layers of the retina, regulating intraocular pressure, blocking light from entering the eye through the sclera, and ensuring clear imaging. Prolonged near-work activities in children and adolescents, which require constant adjustment for clear retinal imaging, lead to reduced blood flow in the choroid and thinning of the sclera due to hypoxia. Eventually, elongation of the eyeball occurs under the influence of intraocular pressure. The environmental factors associated with ocular growth in myopia typically contribute to choroidal thinning (13–17). Furthermore, choroidal thinning occurs non-uniformly across different areas (11, 12, 14). with highly myopic patients experiencing rapid thinning within a short period (13, 15). Overall, these findings support the importance of choroidal thickness as an indicator of ocular growth and development on children. A better understanding of choroidal characteristics can enhance the prediction of myopia occurrence and progression.

Optical coherence tomography (OCT) is a rapidly advancing imaging technology that holds great promise, particularly in the field of *in vivo* detection and imaging of biological tissues. OCT has found extensive applications in ophthalmic clinical diagnostics, allowing for non-contact and non-invasive cross-sectional imaging of microstructural features in living ocular tissues. Its exceptional penetration depth is largely unaffected by the transparency of ocular refractive media (14, 18). OCT offers multiple scanning modalities, enabling visualization of both the anterior and posterior segments of the eye. The use of spectral-domain OCT (SD-OCT) further enhances depth imaging capabilities, facilitating clear visualization and accurate measurement of choroidal thickness (19).

This study aims to find out the values and distribution of choroidal thickness in myopic children, discuss the relationship between different diopter (D) and choroidal thickness in various areas, and explore choroidal thickness as a critical indicator for evaluating the progression of myopia.

## 2. Methods

### 2.1. Study population

A cluster sampling approach was employed to select participants from the ophthalmology clinic of the Second Hospital of Dalian Medical University, including myopic children and emmetropic children aged 6 to 14 years. The history of neurological disorders, developmental delay, amblyopia, other ocular diseases, and poor cooperation during examinations were excluded from the study. All participants and their legal guardians were fully informed about the study protocol and provided informed consent (for children below 8 years of age, consent was obtained from their guardians, while children aged 8 years and above provided their own assent along with consent from their guardians). The study adhered to the principles outlined in the Declaration of Helsinki and obtained ethical approval from the Ethics Review Committee of the Second Hospital of Dalian Medical University.

### 2.2. Study methods

**Figure 1:**
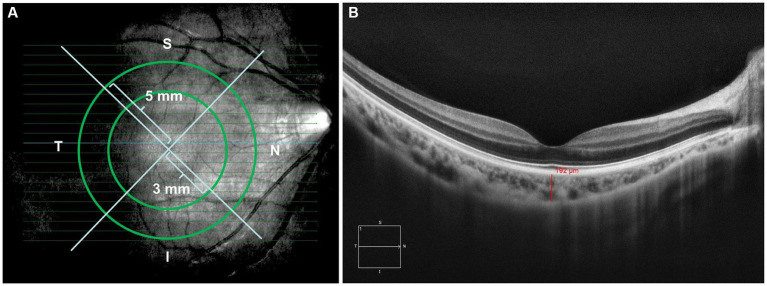
**(A)** Images from (HD 21 line) 360-degree 0 mm, 3 mm, and 5 mm radii peripapillary circle scans; **(B)** examples of images demonstrating manual delineation of the choroidal thickness from the RPE to the choroid-sclera junction.

This is a retrospective study. A total of 156 eyes were collected and divided into four groups: Group 1, Low myopes (40 eyes, −0.25D < SER ≤ −2.75D); Group 2, Moderate myopes (45 eyes, −2.75D < SER ≤ −5.00D); Group 3, High myopes (41 eyes, SER < −5.00D); Group 4, Emmetropes (30 eyes, +0.25D < SER ≤ −0.25D). Demographic data, specifically age and sex, were procured from the electronic medical record system. Ophthalmic examinations were documented encompassing AL, Cycloplegic SER using compound tropicamide, and SD-OCT images captured utilizing the HD 21 Line mode of the Cirrus HD-OCT instrument (Carl Zeiss) to optimize choroidal depth imaging. The images were exported and the ETDRS grid was employed for choroidal thickness mapping. Manual segmentation was performed to acquire images within concentric circles of 0 × 0 mm, 3 × 3 mm, and 5 × 5 mm radii on the macular central fovea (Figure 1A). The areas were classified into central fovea, parafovea, and perifovea, with radii of 0 mm, 3 mm, and 5 mm, respectively, further divided into superior, inferior, nasal, and temporal areas, resulting in nine evenly distributed areas. The built-in ruler plugin in the instrument was used to measure the average choroidal thickness in each area. Choroidal thickness was defined as the distance between the retinal pigment epithelium (RPE) and the choroid-sclera junction (the outermost layer with high reflectivity to low-reflectivity line, Figure 1B) (17, 19–22). Measurements were repeated multiple times by the same examiner, and the average values were recorded. The pediatric OCT images were obtained between 9:00 am and 2:00 pm to reduce the influence of diurnal variations in choroidal thickness (23–26).

### 2.3. Statistical analysis

The statistical analysis was performed using SPSS software version 25.0. The Kolmogorov–Smirnov test was used to assess the normality of all continuous variables. The results indicated a normal distribution, and the values for choroidal thickness in all areas were expressed as mean ± standard deviation (SD) in μm. Independent *t*-tests were used to compare variables between two groups, and one-way analysis of variance (ANOVA) was used for multiple group comparisons. Pearson correlation analysis was conducted, followed by linear regression analysis to examine the relationship between choroidal thickness and SER/AL. A value of *p* <0.05 was considered statistically significant.

## 3. Results

The study included children aged of 10.24 ± 1.89 years (range: 6 to 14 years), with 66 boys and 90 girls. The correlation between age and choroidal thickness was investigated and found to be non-significant (Table 1). The average AL among all children was 23.94 ± 1.24 mm (range: 22.95 to 27.34 mm), and the average SER was −3.42 ± 2.67 D (range: + 0.25 D to −11.00 D). There were 40 cases of low myopes, 45 cases of moderate myopes, 41 cases of high myopes, and 30 cases of emmetropes. The general characteristics of the participating children are presented in Table 2.

**Table 1:** Comparison of choroidal thickness among the three age groups (1–3) ranging from 6 to 14 years.

Choroidal thickness based on area (Mean ± SD) μm	Age groups (D)			
	Group1 = 6–8 years (*n* = 56)	Group2 = 9–11 years (*n* = 59)	Group3 = 12–14 years (*n* = 41)	p*
C = Sub Fovea	230.46 ± 52.699	240.46 ± 34.277	231.63 ± 24.756	0.355
N3 = Nasal 3	213.46 ± 46.789	219.37 ± 30.396	216.80 ± 28.533	0.690
N5 = Nasal 5	201.38 ± 46.328	206.71 ± 30.862	205.12 ± 25.624	0.723
T3 = Temporal 3	232.54 ± 49.136	244.00 ± 35.425	239.32 ± 24.183	0.284
T5 = Temporal 5	241.38 ± 47.514	250.85 ± 32.988	246.90 ± 24.214	0.393
S3 = Superior 3	230.61 ± 53.572	240.36 ± 33.100	232.85 ± 24.040	0.398
S5 = Superior 5	239.73 ± 51.530	249.46 ± 31.521	251.22 ± 23.647	0.262
I3 = Inferior 3	217.16 ± 61.015	229.46 ± 42.053	227.51 ± 32.434	0.349
I5 = Inferior 5	207.50 ± 57.522	220.88 ± 42.937	220.98 ± 32.975	0.229

*Statistically significant difference among the groups. *p* < 0.05 was considered statistically significant.

**Table 2:** Basic information of all children.

	Myopia degree (D)				
	Emmetropes (*n* = 30)	Low myopes (*n* = 40)	Moderate myopes (*n* = 45)	High myopes (*n* = 41)	p*
Age, year	9.39 ± 1.425	9.66 ± 1.744	10.23 ± 1.607	11.44 ± 2.035	^b^0.000
SEX(M/F)	13/17	15/25	22/23	16/25	^a^0.712
SER	0.06 ± 0.204	−1.75 ± 0.700	−4.04 ± 0.725	−6.92 ± 1.241	^b^0.000
AL (mm)	23.48 ± 0.299	24.03 ± 0.336	25.18 ± 0.373	26.64 ± 0.393	^b^0.000

^a^chi-squared test.

^b^One-way Anova test.

*Statistically significant difference among the groups. *p* < 0.05 was considered statistically significant.

**Figure 2:**
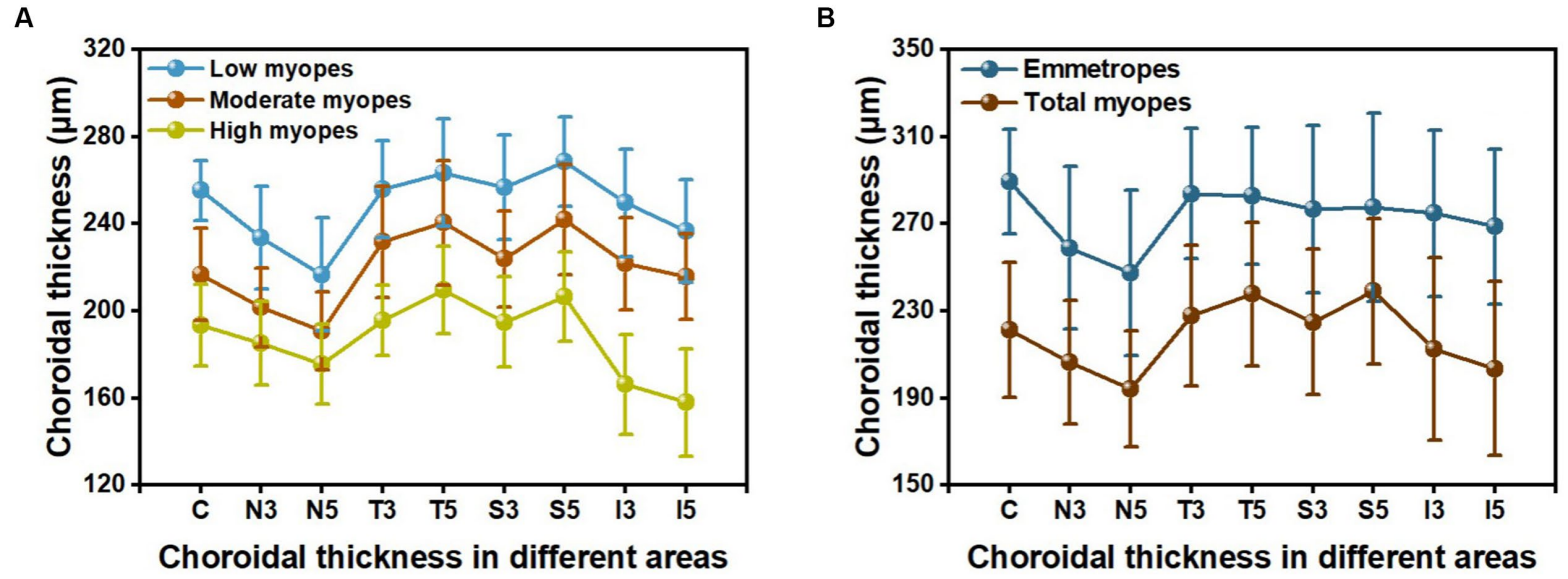
The choroidal thickness in nine areas: **(A)** among three groups of myopic children; **(B)** between emmetropes and myopes.

The choroidal thickness was compared across different groups and areas (Tables 3, 4). Back testing using the least significant difference (LSD) method revealed when −5.00D ≤ SER < −0.25D, the choroidal thickness in the superior area was thicker than the inferior, nasal, and temporal areas, and the thickness in the perifovea was greater than the parafovea in the superior area. The choroidal thickness was highest at the farthest distance from the central fovea in the superior area (S5). In the low myopes and moderate myopes, the choroidal thickness in the superior perifovea (S5) was 268.68 ± 20.52 μm and 242.07 ± 25.19 μm, respectively, while the thinnest thickness was observed in the nasal perifovea (N5) at 216.80 ± 25.99 μm, 190.76 ± 17.96 μm, respectively. In the highly myopic group (SER < −5.00D), the outermost layer of the temporal area (T5) exhibited the thickest choroidal thickness at 209.63 ± 20.27 μm, while the thinnest thickness was observed in the inferior area (I5) at 157.90 ± 24.76 μm (Tables 3, 4; Figure 2).

**Table 3:** Comparison of choroidal thickness between emmetropes and total myopes.

Choroidal thickness based on area (Mean ± SD) μm	Myopia degree (D)		
	Emmetropes (*n* = 30)	Total myopes (*n* = 126)	p*
C = Sub Fovea	289.50 ± 23.893	221.47 ± 30.989	0.000
N3 = Nasal 3	259.03 ± 37.388	206.47 ± 28.295	0.000
N5 = Nasal 5	247.53 ± 38.142	194.10 ± 26.689	0.000
T3 = Temporal 3	283.90 ± 29.924	227.88 ± 32.343	0.000
T5 = Temporal 5	282.93 ± 31.316	237.71 ± 32.905	0.000
S3 = Superior 3	276.80 ± 38.561	224.90 ± 33.358	0.000
S5 = Superior 5	277.67 ± 43.202	238.99 ± 33.426	0.000
I3 = Inferior 3	274.87 ± 38.321	212.55 ± 41.718	0.000
I5 = Inferior 5	268.77 ± 35.490	203.56 ± 39.933	0.000

*Independent *t*-test. *p* < 0.05 was considered statistically significant.

**Table 4:** Comparison of choroidal thickness and degrees of myopia.

Choroidal thickness based on area (Mean ± SD) μm	Myopia degree (D)			
	Low myopes (*n* = 40)	Moderate myopes (*n* = 45)	High myopes (*n* = 41)	p*
C = Sub Fovea	255.35 ± 13.632	216.78 ± 21.235	193.56 ± 18.786	0.000
N3 = Nasal 3	233.63 ± 23.626	201.58 ± 18.177	185.34 ± 19.266	0.000
N5 = Nasal 5	216.80 ± 25.984	190.76 ± 17.962	175.63 ± 18.312	0.000
T3 = Temporal 3	255.75 ± 22.288	231.69 ± 25.643	195.51 ± 16.144	0.000
T5 = Temporal 5	263.38 ± 24.639	240.49 ± 28.692	209.63 ± 20.267	0.000
S3 = Superior 3	256.78 ± 24.114	223.93 ± 22.056	194.88 ± 20.841	0.000
S5 = Superior 5	268.68 ± 20.517	242.07 ± 25.194	206.66 ± 20.485	0.000
I3 = Inferior 3	249.63 ± 24.697	221.80 ± 24.213	166.22 ± 22.984	0.000
I5 = Inferior 5	236.58 ± 23.622	215.82 ± 19.737	157.90 ± 24.759	0.000

*One-way Anova test. *p* < 0.05 was considered statistically significant.

**Figure 3:**
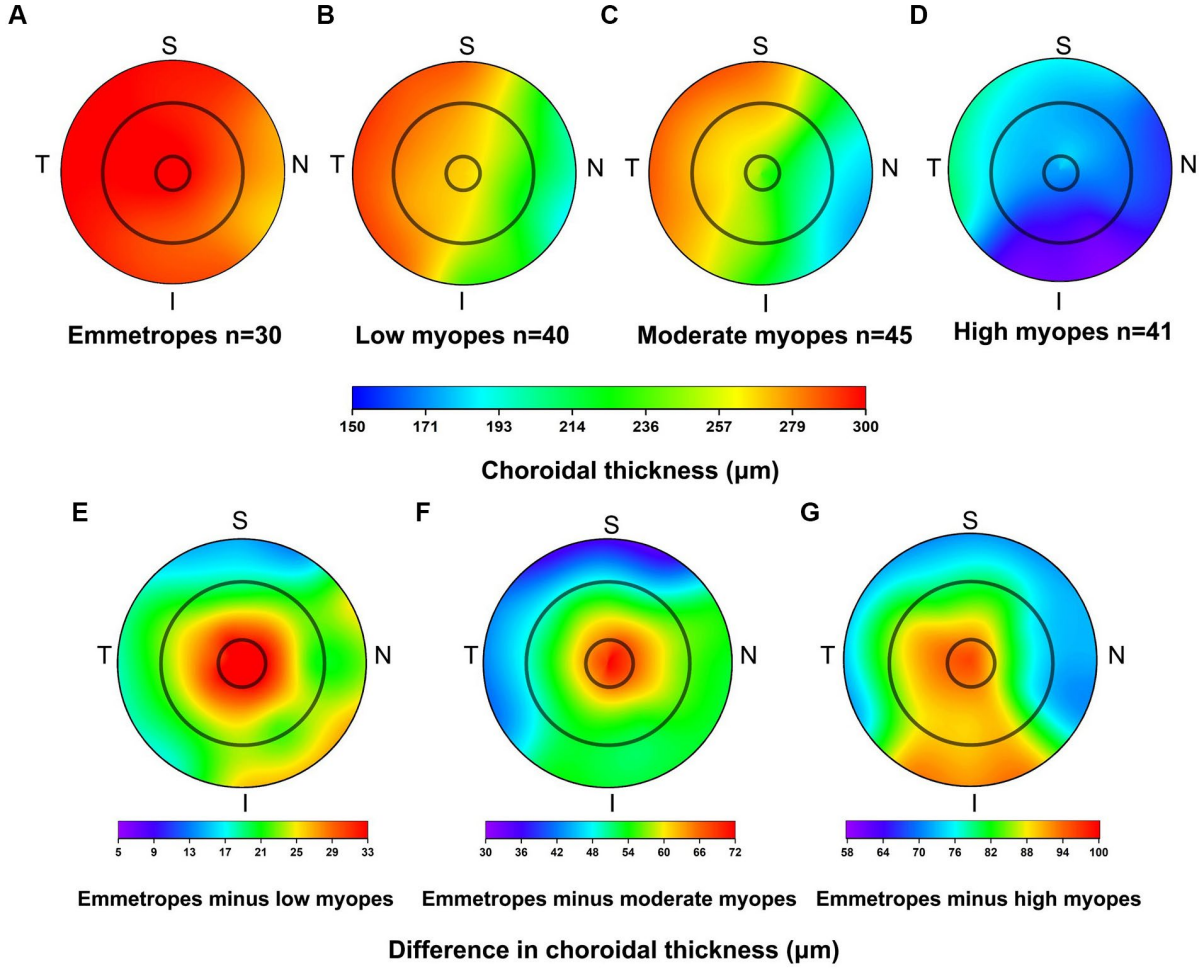
Topographical variation of choroid thickness in emmetropes **(A)**, low myopes **(B)**, moderate myopes **(C)**, high myopes **(D)**. Average difference in choroidal thickness between emmetropes and low myopes **(E)**, emmetropes and moderate myopes **(F)**, and as well as between emmetropes and high myopes **(G)**.

For all participants, the choroidal thickness was mapped according to diopter groups using the ETDRS grid. From Figure 3, it can be observed that as the degree of myopia increases, the choroidal thickness in all areas shows a decreasing trend. The children in the emmetropic group have the thickest choroid, while the highly myopic group has the thinnest choroid (*p* < 0.01). In the horizontal direction, the choroidal thickness of all myopic children decreased from the temporal to the nasal areas (T5 > T3 > C > N3 > N5, all *p* < 0.01, Table 4). In the vertical direction, there were variations in choroidal thickness among different degrees of myopia. Further data analysis revealed that when SER ≥ −2.75D, the choroidal thickness in the perifoveal areas is smaller than that in the parafoveal areas, i.e., the average thickness of S5 and I5 is smaller than that of S3 and I3 (all *p* < 0.01; Figure 3B; Table 5). When SER < −2.75D, the choroidal thickness in the more peripheral areas is greater than that in the parafoveal areas (all *p* < 0.01, Figures 3C,D; Table 5). The difference in choroidal thickness between each myopia group and the emmetropic group was calculated for different areas, and the average difference values were used to generate choroidal thickness difference maps (Figures 3E–G). It was found that the closer the area is to the central fovea, the greater the difference in choroidal thickness, while the closer it is to the perifovea, the smaller the difference. The difference in choroidal thickness in the superior area is smaller than that in the lower, temporal, and nasal areas.

**Table 5:** Choroidal thickness 3 mm and 5 mm from the Sub Fovea in the vertical direction.

Choroidal thickness (Mean ± SD) μm	Myopia degree (D)			
	Low myopes (*n* = 80)	Moderate myopes (*n* = 90)	High myopes (*n* = 82)	p*
S3 + I3	253.20 ± 24.517	222.87 ± 23.054	180.55 ± 218.73	0.000
S5 + I5	252.63 ± 27.279	228.94 ± 26.087	182.28 ± 33.353	0.000

*One-way Anova test. *p* < 0.05 was considered statistically significant.

**Figure 4:**
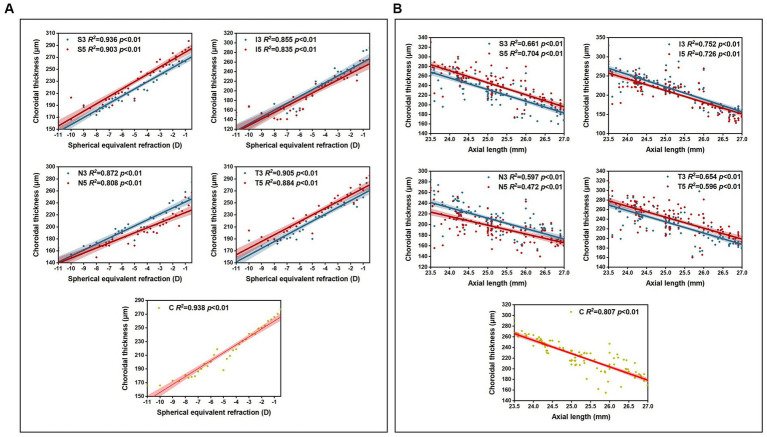
The relation between CT and SER **(A)**/AL **(B)**.

Pearson correlation analysis revealed significant correlations (*p* < 0.01) between choroidal thickness in the nine areas and SER/AL, and linear regression analysis was conducted (Table 6). The results showed that in all myopic children, SER was positively correlated with choroidal thickness in all areas (all *p* < 0.01), with the strongest correlation observed between SER and the inferior area (I3 + I5) (*β* = 14.85; *R^2^* = 0.68; *p* < 0.01), and a stronger correlation between SER and I3 (*β* = 15.40; *R^2^* = 0.71; *p* < 0.01). AL was negatively correlated with choroidal thickness in all areas (all *p* < 0.01), with the strongest correlation observed between AL and the inferior area (I3 + I5) (*β* = −31.34; *R^2^* = 0.73; *p* < 0.01), and a stronger correlation between AL and I3 (*β* = −32.41; *R^2^* = 0.75; *p* < 0.01). Furthermore, the analysis of choroidal thickness in the central fovea revealed that for every 1 D increase in myopia, the subfoveal choroidal thickness decreased by 13 μm, and for every 1 mm increase in AL, the subfoveal choroidal thickness decreased by 23 μm (Figure 4).

**Table 6:** Simple linear regression results between choroidal thickness and spherical equivalent refraction and axial length.

Area	SER and choroidal thickness			AL and choroidal thickness		
	*r* (correlation)	Adjusted *R^2^*	p*	*r* (correlation)	Adjusted *R*^2^	p*
C = Sub Fovea	0.969	0.938	0.000	−0.899	0.807	0.000
N3 = Nasal 3	0.936	0.872	0.000	−0.775	0.597	0.000
N5 = Nasal 5	0.902	0.808	0.000	−0.690	0.472	0.000
T3 = Temporal 3	0.908	0.905	0.000	−0.810	0.654	0.000
T5 = Temporal 5	0.942	0.884	0.000	−0.774	0.596	0.000
S3 = Superior 3	0.968	0.936	0.000	−0.815	0.661	0.000
S5 = Superior 5	0.952	0.903	0.000	−0.841	0.704	0.000
I3 = Inferior 3	0.927	0.855	0.000	−0.868	0.752	0.000
I5 = Inferior 5	0.916	0.835	0.000	−0.853	0.726	0.000

*Linear regression analysis. *p* < 0.05 was considered statistically significant.

## 4. Discussion

In this study, the choroidal thickness showed a characteristic distribution pattern and different diopters had different effects on the choroidal distribution (*p* < 0.01), which is consistent with previous research (20–23, 27–31). Song et al. studied choroidal thickness in 82 patients with high myopia (15), the results showed that the thickest choroid was observed in the temporal periphery (192.31 ± 25.39 μm), while the thinnest choroid was found in the inferior periphery (154.45 ± 25.37 μm). Furthermore, as there were differences in the distribution pattern of choroidal thickness between high myopes and low to moderate myopes, this study bridged the gap in research on choroidal thickness distribution in children with low to moderate myopia. For low to moderate myopic children, the thickest choroid was observed in the superior periphery, while the thinnest choroid was found in the nasal periphery.

In a report by Xiong et al. (14), which examined the choroidal thickness of 3,001 Chinese children aged 6–19 years, they investigated whether there were differences in choroidal thickness among different regions (based on the ETDRS grid division), and found that in the vertical direction, the choroid in the parafovea was thicker than the choroid in the perifovea for all myopic children. Building upon this, our study further discussed the subdivision based on diopter in myopic children. Among children with low myopia (SER ≥ −2.75D), the results were consistent with Xiong et al.’s findings, whereas in children with moderate to high myopia (SER < −2.75D), the choroid in the perifovea was thicker. In agreement with Xiong et al.’s report, our study found that myopic children had the greatest difference in choroidal thickness (central fovea: mean difference = 68.03 μm, parafovea: mean difference = 55.70 μm, perifovea: mean difference = 50.64 μm). However, these differences were different from their report, possibly due to measurement errors, different OCT light sources, and sex differences. Given the limited research on the difference in choroidal thickness, and the limitations in sample size in our study, this conclusion requires further longitudinal studies for validation.

The correlation between AL and SER with choroidal thickness has been demonstrated in previous studies (28). In our study, we found a positive correlation between CT and SER, and a negative correlation between CT and AL. Among these correlations, the thickness variation in the perifovea of the inferior area was most influenced by SER and AL. However, the reason for this phenomenon is unclear. We speculated that this difference was due to uneven eye development, as supported by embryological studies. The optic fissure located below the optic cup, which is the last part to close during eye development. Consequently, the inferior area experiences greater vascular resistance, making it more susceptible to changes in other factors, resulting in variations in choroidal thickness (30). Lee et al. have reported that myopic patients often present with an uneven thinning of the choroid, with the choroid in the temporal area being thicker than the fovea (32). And many studies indicated asymmetries and eccentricity-dependent differences in the pattern of ocular growth (13). In patients with higher levels of myopia, a thinner choroidal thickness may affect photoreceptor function, leading to decreased vision. At the same time, there may be a number of complications, such as posterior scleral staphyloma and lacquer cracks in or near the macular area. In addition, thinner choroid in myopic patients also show a strong positive association with the severity of myopia traction maculopathy. Therefore, monitoring choroidal thickness may provide useful clues to identify those at risk of faster eye growth, as well as those at risk of high myopia-related pathology.

The relationship between age and choroidal thickness remains highly controversial, but most researchers believe that statistical analysis should be conducted within a smaller age range to obtain more generalizable conclusions (17, 31, 33). Our study was based on a smaller age range of participants, demonstrated the uneven growth of choroidal thickness during ocular development, but age was not found to be a significant influencing factor (*p* > 0.05) for choroidal thickness.

According to previous experimental animal models, including macaque monkeys, guinea pigs, and chicks, it has been found that changes in choroidal thickness precede AL changes. AL changes often serve as a rapid compensation for choroidal thickness to optimize imaging quality (34–37). Similarly, based on previous studies on refractive development theory, the choroid is the first to thin rapidly and act on the sclera when the imaging is not clear. Subsequently, the extracellular matrix was induced by this mechanism to reshape, which directly affect eye growth (38–43). In the future advanced studies, the sequence of changes in choroidal thickness and other myopia indicators should be studied and analyzed. Nickla et al. cultivated white leghorn chicks to establish an animal model, and the research results revealed that nighttime light can cause diurnal variations in choroidal thickness. Specifically, the AL is longer during the day, resulting in a thinner choroid (26), while at night, the AL becomes shorter, leading to a thicker choroid (23, 24, 26). Therefore, in order to minimize the influence of diurnal variations on choroidal thickness, this study selected a specific time period (9–14 h) for measuring choroidal thickness. Based on the findings of this study, further research on the diurnal variations of choroidal thickness will be conducted, which will contribute to a better understanding of the correlation between myopia and the choroid.

The limitations of this study include the manual measurement of choroidal thickness, which introduces errors, as well as the limited sample size. Although the results of this study revealed a correlation between choroidal thickness and myopia, the study participants were relatively young, and the compliance was slightly poor, which may have resulted in biases in the research results. In our future studies, further examinations will be conducted to address the limitations in terms of sample size and participant demographics.

## 5. Conclusion

In conclusion, this study analyzed the choroidal thickness in pediatric OCT images and demonstrated that choroidal thickness is a valuable indicator for evaluating myopia in children. The results showed that the perifoveal choroid was thicker than the parafoveal choroid in children with moderate to high myopia (SER < −2.75D), which was different from previous reports. Meanwhile, SER and AL had the greatest influence on the choroidal thickness in the inferior area. Furthermore, this study bridged the gap in the study of choroidal thickness distribution in children with low and moderate myopia. Therefore, considering the urgent need for myopia control in children, based on the differences observed in choroidal thickness, we strongly recommend the widespread use of choroidal examination in myopic patients to prevent a range of serious development of myopia.

## Data availability statement

The original data presented in the study is included in the article, further inquiries can be directed to the corresponding author.

## Ethics statement

The studies involving humans were approved by the Ethics Review Committee of the Second Hospital of Dalian Medical University. The studies were conducted in accordance with the local legislation and institutional requirements. Written informed consent for participation was not required from the participants or the participants’ legal guardians/next of kin in accordance with the national legislation and institutional requirements.

## Author contributions

HZ and QZ made substantial contributions to design of the work and revise the manuscript critically, drafted the manuscript, and agreed to be accountable for all aspects of the work. CL and MG contributed to data collection. SZ and LZ contributed to conduct the statistical analysis and draw diagrams. All authors have contributed to the final manuscript for important intellectual and read and approved the final manuscript.
